# 

**Published:** 1911-03

**Authors:** 


### BOOKS RECEIVED

---

The Principles and Practice of Modern Otology. By John F. Barnhill, M.D., Professor of Otology, Laryngology, and Rhinology, Indiana University School of Medicine; and Ernest deW. Wales, B.S., M.D., Clinical Professor of Otology, Laryngology and Rhinology, Indiana University School of Medicine. Second edition revised. Octavo of 598 pages, with 305 original illustrations, many in colors. Philadelphia and London: W. B. Saunders Company. 1911. (Cloth, $5.50; half morocco, $7.00, net prices.)

Personal Hygiene and Physical Training for Women. By Anna M. Galbraith, M.D., Fellow of the New York Academy of Medicine. 12 mo of 371 pages, with original illustrations. Philadelphia and London: W. B. Saunders Company. 1911. (Cloth, $2.00, net.)

State Registration for Nurses. By Louie Croft Boyd, R. N. Graduate of Colorado School for Nurses. 12 mo of 42 pages. Philadelphia and London: W. B. Saunders Company. 1911. (Price, 50c net.)

The Practical Medicine Series. Ten Volumes. Under the general editorial charge of Gustavus P. Head, M.D. Vol. X. Nervous and Mental Diseases. Edited by Hugh T. Patrick, M.D., and Peter Bassoe, M.D. Series 1910. Chicago: The Year Book Publishers. (Price, $1.25; entire series, $10.00.)

Diseases of Children for Nurses. Including Infant Feeding, Therapeutic Measures Employed in Childhood, Treatment for Emergencies, Prophylaxis, Hygiene and Nursing. By Robert S. McCombs, M.D., Assistant Physician to the Dispensary and Instructor of Nurses at the Children's Hospital of Philadelphia. Second Edition Revised. Octavo of 470 pages. Illustrated. Philadelphia and London: W. B. Saunders Company. 1911. (Cloth, $2.00, net.)

Collected Papers by the Staff of St. Mary's Hospital, Mayo Clinic, Rochester, Minnesota. 1905-1909. Octavo of 668 pages. Illustrated. Philadelphia and London: W. B. Saunders Company. 1911. (Cloth, $5.50, net.)

Differential Diagnosis. Presented through an Analysis of 383 cases. By Richard C. Cabot, M. D., Assistant Professor of Clinical Medicine, Harvard Medical School. Octavo of 753 pages. Illustrated. Philadelphia and London: W. B. Saunders Company. 1911. (Cloth, $5.50, net.)

A Handbook of Practical Treatment. In three volumes. By 79 eminent specialists. Edited by John H. Musser, M.D., Professor of Clinical Medicine, University of Pennsylvania; and A. O. J. Kelly, M.D., Assistant Professor of Medicine, University of Pennsylvania. Volume I. Octavo of 909 pages. Illustrated. Philadelphia and London; W. B. Saunders Company. 1911. (Per volume, cloth, $6.00, half morocco, $7.50, net prices.)

Systemic (including Special) Pathology. By J. George Adami M.A., M.D., LL.D., F.R.S., Professor of Pathology, and Albert G. Nicholls, M.A., M.D., F.R.S., Assistant Professor of Pathology in McGill University, Montreal. In one octavo volume of 1,160 pages with 301 engravings and 15 plates. Philadelphia and New York: Lea & Febiger. 1911. (Cloth, $6.00, net.)

Atlas of Microscopic Diagnosis in Gynecology. By Dr. Rudolf Jolly, Berlin. Translated by P. W. Shedd, M.D., New York. Royal octavo, pp. 192. With 52 lithographs in color and 2 textual figures. New York: Rebman Co. (Cloth, $5.50.)

Makers of Man. By Charles J. Whitby, M.D., author of "The Logic of Human Character." Octavo, pp. 424. Illustrated. New York: Rebman Co. (Cloth, $3.00.)

Case Histories in Pediatrics. By John Lovett Morse, M.D., Assistant Professor of Pediatrics, Harvard Medical School. Octavo, pp. 314. Boston: W. M. Leonard. (Cloth, $3.00.)

Handbook for Treatment of Diseases of the Eye. By Dr. Curt Adam, Berlin. Translated by William George Sym, M.D., and E. M. Lithgow, M.B. 12 mo., pp. 276. Illustrated. New York: Rebman Co. (Cloth, $2.50.)

Plastic and Cosmetic Surgery. By Frederick Strange Kolle, M.D., author of "The x-rays; their Production and Application." Octavo, pp. 532. Illustrated. New York and London: D. Appleton & Co. 1911. (Cloth, $5.00.)

Memoranda on Poisons. By Thomas H. Tanner, M.D. Eleventh revised edition by Henry Leffmann, M.D. Philadelphia: P. Blakiston's Son & Co. (Cloth, 75 cents.)

Transactions of the Fourth International Sanitary Conference of the American Republics, held at San José, Costa Rica, December 25, 1909, to January 3, 1910.

Modern Treatment: The Management of Disease with Medicinal and Non-Medicinal Remedies. By Eminent Amercian and English Authorities. Edited by Hobart Amory Hare, M.D., Professor of Therapeutics and Materia Medica, Jefferson Medical College, Philadelphia, assisted by H. R. M. Landis, M.D. Medical Director to the Phipps Institute for Tuberculosis and Physician to the White Haven Sanatorium. In two octavo volumes, comprising 1,800 pages, with numerous engravings and full page plates. Lea and Febiger, Publishers, Philadelphia and New York, 1911. (Price per volume in cloth, $6.00; half morocco, $7.50, net.)

The Blues; Causes and Cures. By Albert Abrams, A.M., M.D., Late Professor of Pathology and Director of the Medical Clinic, Cooper Medical College, San Francisco, Cal. Fourth Edition. 8vo. 304 pages. Illustrated. E. B. Treat & Co., New York. (Cloth, $1.50.)

